# Complete genome sequence of *Serratia plymuthica* PAMC22778 from Kara sea sediment reveals biosynthetic gene clusters with potential biocontrol-associated secondary metabolites

**DOI:** 10.1128/mra.00244-26

**Published:** 2026-06-24

**Authors:** Paras Paudel, Byeollee Kim, Sang-Hee Jung, Yung Mi Lee, Jun Hyuck Lee, Tae-Jin Oh

**Affiliations:** 1 Department of AI Bio-Big Data Convergence, Graduate School SunMoon University, Asan, South Korea; 2 Genome-based BioIT Convergence Institute, Asan, South Korea; 3 Department of Dental Hygiene, Gangneung Yeongdong University 357131, Gangneung, South Korea; 4 Division of Life Sciences, Korea Polar Research Institute 123591, Incheon, South Korea; 5 Department of Pharmaceutical Engineering and Biotechnology, SunMoon University 35022 , Asan, South Korea; Montana State University, Bozeman, Montana, USA

**Keywords:** *Serratia *sp., complete genome, biosynthetic gene cluster, biocontrol agent

## Abstract

We report the complete genome of *Serratia plymuthica* PAMC22778, a soil-associated bacterium with biocontrol potential. The complete genome comprises 5,765,014 bp, including one circular chromosome of 5,661,166 bp and one plasmid of 103,848 bp, with a GC content of 55.54%, and encodes biosynthetic gene clusters for siderophores, prodigiosin, and biosurfactants, implicated in iron acquisition and antimicrobial activity, highlighting its potential as a biocontrol agent.

## ANNOUNCEMENT

The genus *Serratia* comprises metabolically versatile bacteria commonly associated with diverse environments (1). Members of this genus exhibit diverse physiological and biochemical capabilities, immensely recognized for their biocontrol and plant growth-promoting properties (2, 3). Despite the importance of this genus, few studies have characterized *Serratia plymuthica*, a recently described species, limiting our understanding of its ecological role and biotechnological potential (4).

Here, we present the complete genome sequence of Serratia plymuthica PAMC22778 (PAMC22778), isolated from sediment, Kara Sea. The strain PAMC22778 was deposited by Korea Polar Research Institute. For taxonomic classification, PAMC22778 was cultured on R2A at 15°C for 3 days (5). Pure culture on the plate was used for DNA extraction using the DNeasy Blood & Tissue Kit (Qisgen, Netherlands) following the manufacturer’s methods. The sequencing was performed by PHYZEN Co. (Seongnam-si, Korea). PAMC22778 was sequenced using both Oxford Nanopore Technologies (ONT) (6) and Illumina platforms. Illumina library preparation used the TruSeq DNA Nano Kit (Illumina, USA) without size selection, and sequencing was performed on a NovaSeq 6000 platform (2 × 150 bp paired-end reads). ONT libraries were prepared without DNA shearing or size selection using the Native Barcoding Expansion Kit (EXP-NBD104) and 1D Ligation Sequencing Kit (SQK-LSK110). GridION sequencing used FLO-MIN106 (R9.4) flow cells with high-accuracy basecalling (HAC mode), Illumina yielded 8,587,983 short reads comprising 1,296,612,472 bases, which were quality-filtered and adapter-trimmed using fastp and subsequently used for polishing the assembly, while ONT sequencing generated 730,005,621 bp of high-quality long-read data (121× coverage, read N50 of 18.5 kb, and N90 of 5.7 kb). Long reads were assembled using Flye v2.9.3 (6) and reconciled using Trycycler v0.5.4 (7). The assembly was polished using Medaka v1.11.3, Polypolish v.06.0, and POLCA from MaSuRCA v4.1.0 was improve consensus accuracy. The final polished assembly showed 97.4% and 92.4% of bases with quality scores above Q20 and Q30, respectively.

The resulting complete genome comprised 5,765,014 bp, consisting of one circular chromosome (5,661,166 bp) and one plasmid (103,848 bp), with a GC content of 55.54%. Genome completeness was estimated at 96.71% using CheckM with a contamination level of 1.57% (8). Annotation was performed with Prokka v1.14.6 (9), eggnog-mapper v2.1.8 (10), and RAST (11), which identified 5,341 coding sequences, 85 tRNAs, 22 rRNAs, one tmRNA, and complete rRNA operons (5S, 16S, and 23S). Average nucleotide identity analysis based on OrthoANI by Ezbiocloud revealed a genomic similarity of 96.79% with the reference genome of *Serratia plymuthica* (12). For all software, default parameters were used unless otherwise specified. The genome features of PAMC22778 are presented in Table 1.

**TABLE 1:** Summary of genome information of Serratia *plymuthica* PAMC22778

Parameters	Genomic features
ONT statistics	
Total number of base pairs	730,055,621
Genomic coverage	121×
High-quality reads N50	18.5 kb
High-quality reads N90	5.7 kb
Assembly size	5.76 Mb
Genome	
Size (bp)	5,765,014 bp
GC content (%)	55.54%
Completeness	96.71%
Contamination	1.57%
Contig N50 (bp)	5,661,166 bp
Contig L50	1
Protein-coding genes	5,341
tRNA	85
Highest 16S identity	99.74%
Highest ANI (OrthoANI)	96.79% with *Serratia plymuthica*

Secondary metabolite biosynthetic gene clusters (BGCs) were identified using antiSMASH v8.0.2 (13) with default parameters, including ClusterBlast and KnownClusterBlast analyses. The genome harbors at least 15 predicted BGCs, including arylpolyene-siderophore hybrid cluster (region 2.3), NRPS-metallophores (regions 2.4 and 2.12), prodigiosin clusters (region 2.8), and zeamine-like clusters (region 2.14). All these findings suggest that PAMC22778 may be a promising candidate for a biocontrol agent.

## Data Availability

The genome of *Serratia plymuthica* PAMC22778 has been submitted by the European Nucleotide Archive (ENA) under study accession number PRJEB106064 and sample accession number SAMEA121008655. The raw sequencing reads have been deposited to the ENA under accession numbers ERX15535197 and ERX16402859. GenBank accession number was OZ470281.1. The complete genome sequence of *Serratia plymuthica* PAMC22778 has been deposited in GenBank under accession number GCA_978045405.1.
